# Calligraphed Selective Plasmonic Arrays on Paper Platforms for Complementary Dual Optical “ON/OFF Switch” Sensing

**DOI:** 10.3390/nano10061025

**Published:** 2020-05-27

**Authors:** Laurentiu Susu, Andreea Campu, Simion Astilean, Monica Focsan

**Affiliations:** 1 Nanobiophotonics and Laser Microspectroscopy Center, Interdisciplinary Research Institute on Bio-Nano-Sciences, Babes-Bolyai University, Treboniu Laurean No. 42, 400271 Cluj-Napoca, Romania; susulaurentiu@yahoo.com (L.S.); andreea.campu@gmail.com (A.C.); simion.astilean@phys.ubbcluj.ro (S.A.); 2 Biomolecular Physics Department, Faculty of Physics, Babes-Bolyai University, M Kogalniceanu No. 1, 400084 Cluj-Napoca, Romania

**Keywords:** nanoparticles, plasmonic lines, multiplexed paper substrate, ON/OFF switch detection, SERS, MEF

## Abstract

Designing innovative (nano)detection platforms, respecting their low-cost and fabrication simplicity, capable to chemically detect multiple target analytes by employing the same engineered device, is still a great challenge in the multiplexed biosensor development. In this scientific context, in the current manuscript, we exploit the low-cost plasmonic calligraphy as a versatile approach to directly draw continuous plasmonic lines on Whatman paper using a regular ballpoint pen successively filled with two different anisotropic nanoparticles shapes (gold bipyramids—AuBPs and gold nanorods—AuNRs) as colloidal inks. After the efficient immobilization of the positively-charged AuBPs and AuNRs onto the paper fibres, proved by Scanning Electron Microscopy (SEM) investigations, the specificity of our as-calligraphed-paper platform is ensured by coating the selected lines with a thin layer of anionic poly(styrene sulfonate) polyelectrolyte, creating, consequently, a well-defined plasmonic array of charge-selective regions. Finally, the functionality of the well-isolated and as-miniaturized active plasmonic array is, subsequently, tested using the anionic Rose-Bengal and cationic Rhodamine 6G target analytes and proved by complementary dual optical “ON/OFF Switch” sensing (i.e. Surface-enhanced Raman Scattering sensing/metal-enhanced fluorescence sensing) onto the same plasmonic line, developing thus a simple multiplexed plasmonic array platform, which could further facilitate the well-desired biomarker detection in complex mixtures.

## 1. Introduction

Much attention in the scientific community has been continuously paid towards developing efficient (nano)sensing platforms with distinct chemical functionalities and, implicitly, with spatial multiplexed capabilities, that can be effectively transferred to healthcare applications, such as clinical diagnostics and trace sensing detection [1,2].

Noble metal-based nanostructures exhibit surface plasmon resonance which facilitates a phenomenon well-known as Surface-enhanced Raman Scattering (SERS), in which the scattering cross section of the target analytes in contact or very close proximity of metal surface is dramatically increased [3], allowing, consequently, the unique molecular identification of one or multiple “fingerprints” [4,5] at very low concentrations [6,7]. Moreover, considering that the SERS signals present narrow and unique vibrational bands with minimal overlap, SERS can be successfully implemented as a label-free ultrasensitive tool for multiplex detection capable to discriminate different target analytes [8]. Fluorescence spectroscopy is another popular spectroscopic method playing a major role in selective detection of bioanalytes [9]. Actually, the fluorescence signal is quenched when molecules are in contact with metal due to energy transfer from fluorophore to metal and, on the other side, the SERS signal vanishes drastically with the distance from the metal. However, spacing the analyte at an optimal distance (slightly away from the metal) is not detrimental to fluorescence, on the contrary, fluorescence can be enhanced by increasing the excitation and radiative emission rate of the fluorophore in a high-intensity local field, generating the so-called metal- enhanced fluorescence (MEF) phenomenon [10,11,12,13]. The MEF is most efficient when the surface plasmon resonance overlaps with the absorption or emission band of the fluorophore. As SERS and MEF are supported by the excitation of the surface plasmon resonance, to make operational a dual SERS-MEF detection by employing a unique plasmonic platform, it is essential that the platform exhibits distinct surface plasmon resonance bands for each method. Particularly, plasmonic nanoparticles of anisotropic shape, such as gold nanorods (AuNRs) and gold nanobipyramids (AuBPs) are known to exhibit two well-resolved bands, namely transversal and longitudinal resonance bands. Actually, AuNRs and, more recently, AuBPs are excellent SERS-active substrates, due to their strong locally enhanced electromagnetic field at their ends, generating intrinsic “hot-spots”, which leads to the amplification of the Raman signal without inducing aggregation [14,15]. It is expected that such plasmonic nanoparticles can play also as efficient optical (nano)antennas able to enhance the emission rate of the fluorophore with an optimal overlapping between the absorption band and the transversal plasmon resonance band [16], but one of the difficulties in real-world chemical trace detection that must be overcome, is the intrinsic poor chemical selectivity of the plasmonic nanostructures to capture the target analytes from complex (bio)chemical samples [6,17].

Owing to their numerous advantages such as high specific area, flexibility and low-cost, paper substrates are receiving increased attention as efficient and ultrasensitive 3D SERS platforms [18,19]. Cellulose paper is an inexpensive, abundant and biocompatible material, which allows the easy functionalization and absorption of fluids, compared to conventional substrates such as glass or plastic, due to its 3D porous matrix and hydrophilic nature [20]. Therefore, paper-based plasmonic nanoplatforms represent the best compromise in terms of efficiency, cost, and the fabrication process simplicity. Additionally, quantitative results can be obtained using portable spectroscopic techniques as well as commercial cameras and image processing programs [21]. Compared to other substrates, paper can generate a much higher SERS signal due to its micro- and nano-fibre network, which allows the absorption of a larger nanoparticles volume, and, consequently, the detection of a low target analyte concentration [22]. There are numerous methods to immobilize Au nanoparticles on the cellulose fibres, such as plasmonic ink-jet printing, photolithography or wax printing, but these approaches require expensive, sophisticated and, often, time-consuming equipment [23,24]. The most frequently used method for developing paper-based nanoplatforms is through the immersion approach, which is an easy, simple and rapid process with high sensitivity and good reproducibility [25]. Despite this strategy’s many advantages for the fabrication of paper plasmonic substrates, the lack of spatial multiplexing is considered as its main drawback, thus allowing the detection of only a single specific analyte on the test domain. Contrarily, the inexpensive and simple pen-on-paper approach has previously proved its interesting capabilities for multiplex detection [26] or in terms of sensitivity, efficiency, and reproducibility for promoting multimodal biodetection via LSPR/SERS/MEF [27]. 

Therefore, in this study, we design a miniaturized and multiplexed paper-based plasmonic array nanoplatform, using the plasmonic calligraphy approach, by directly writing—using a commercial pen—four separate chemically active plasmonic lines, comprising AuNRs and AuBPs (as colloidal inks) onto the same Whatman no. 1 paper support. Further on, with the aim to demonstrate the functionality and the multiplexed behavior of the as-calligraphed plasmonic platform, one AuBPs and AuNRs plasmonic lines were coated with a layer of negatively-charged poly(styrene sulfonate) (PSS), thus obtaining different charge-selective plasmonic regions. Finally, the “proof-of-concept” multiplex capability of the as-calligraphed plasmonic platform—as smart dual optical “ON/OFF Switch” sensor was validated-in a complementary manner-by the specific Raman fingerprint detection of the captured anionic Rose Bengal (RB) or cationic Rhodamine 6G (R6G) molecules via SERS and the sensitive detection of their fluorescence via the MEF sensing technique. Specifically, by taking advantage of the different charge-selective plasmonic regions, the anion or cationic target molecules were specifically captured and exposed to a huge electromagnetic field generated at the NP’s ends, hence proving the following: (i) when the target analytes are electrostatically attached to the end of the NPs, an efficient SERS detection was obtained by exciting the longitudinal Localized Surface Plasmon Resonance (LSPR) band using a portable 785 nm laser line, along with an implicit effective metal-induced energy transfer (i.e. fluorescence quenching)—due to their close contact with the metal; a better performance of the calligraphed AuBPs line as SERS sensing (nano)antennas compared to the calligraphed AuNRs line was noticed; (ii) when the target analytes were electrostatically repulsed by the NP’s surface, the SERS detection was compromised, but the distance from the metal was still operational to generate MEF by exciting both the transversal LSPR of the NPs and electronic absorption band of the dyes; anew, a different MEF detection performance between these two types of elongated NPs was observed, the AuNRs proving a superior MEF efficiency by recording a maximum of 7.63-fold enhancement for R6G and a 4.96-fold enhancement for RB. Finally, this engineered dual optical “ON/OFF Switch” (nano)sensor, can be extended in the future to detect multiple markers using a single and portable paper-based nanoplatform. 

## 2. Materials and Methods 

### 2.1. Chemicals 

Tetrachloroauric acid (HAuCl_4_·4H_2_O, 99.99%), cetyltrimethylammonium bromide (CTAB, 96%), cetyltrimethylammonium chloride (CTAC), L-ascorbic acid (C_6_H_8_O_6_, 99%), citric acid (C_6_H_8_O_7_), hydroxyquinoline (C_9_H_7_NO, HQL, 99%), sodium borohydride (NaBH_4_, 99%), silver nitrate (AgNO_3_, 99%), nitric acid (HNO_3_), poly(styrene sulfonate) (PSS), Rhodamine 6G (R6G), Rose Bengal (RB) and Whatman^®^ qualitative filter paper, Grade 1 (Whatman no. 1) were purchased from Sigma-Aldrich (St. Louis, MO, USA). Ultrapure water (resistivity ~18.2 MΩ) was used as solvent throughout the experiments.

### 2.2. Synthesis of Anisotropic Gold Nanoparticles in Aqueous Solution 

Gold nanorods (AuNRs) with longitudinal LSPR at 720 nm (denoted as AuNRs@720) were synthesized using the two step seed-mediated approach proposed by Nikoobakht et al. [28]. Briefly, a seed solution was prepared under vigorous stirring by mixing 0.4 M CTAB surfactant with 1 mM HAuCl_4_ and 10 mM NaBH_4_ reducing agent solution. 20 µL of the obtained gold seeds were added to a growth solution, consisting of 0.2 M CTAB, 1 mM HAuCl_4_, 4 mM AgNO_3_ and 78.8 mM ascorbic acid during mild stirring. The mixture was left undisturbed at room temperature until it stabilized.

Gold bipyramids (AuBPs) with longitudinal LSPR located at 753 nm (AuBPs@753) were synthesized using an adapted version of the previously reported seed-mediated growth approach [29]. 1 M HAuCl_4_ with 25 wt % CTAC solution, 0.25 M HNO_3_, 50 mM NaBH_4_ as reducing agent and 1 M citric acid were mixed under vigorous stirring. The final mixture was thermally treated at 80 °C for 60 to 90 min for the formation of the gold seeds, which were then added to a growth solution of 25 mM HAuCl_4_, 45 mM CTAB stabilizing agent solution, 5 mM AgNO_3_ and of 0.4 M HQL as reducing agent. The final solution was left undisturbed at 45 °C for 50 min.

The as-synthesized Au nanoparticle solutions were purified twice by centrifugation followed by redispersion in ultra-pure water for further use.

### 2.3. Preparation of the Calligraphed Plasmonic Paper Platforms 

Commercially available ballpoint (Schneider, Schramberg-Tennenbronn, Germany) and calligraphy (Herlitz, Hanover, Germany) pens with empty refillable cartridges, were bought from a local library store. Concentrated solutions of AuNRs and AuBPs were injected into separate pen cartridges, which were then used to draw four 1 mm wide plasmonic lines, two of each nanostructure, onto a Whatman no. 1 paper strip resulting into four spatially isolated plasmonic regions. The lines were retraced multiple times in the same position in order to increase the optical density of the nanoparticles, and implicitly- to enhance the immobilized nanoparticles concentration onto the porous Whatman paper, thus optimizing the efficiency of the nanoplatform for further experiments. A detailed immobilization protocol of the nanoparticles by simply manipulation of the optical density on paper is thoroughly studied and described in our previously published article [27]. After each trace, the plasmonic substrate was left to dry at room temperature to avoid damaging the paper fibers.

### 2.4. Preparation of Charged Isolated Plasmonic Lines 

**Scheme 1:**
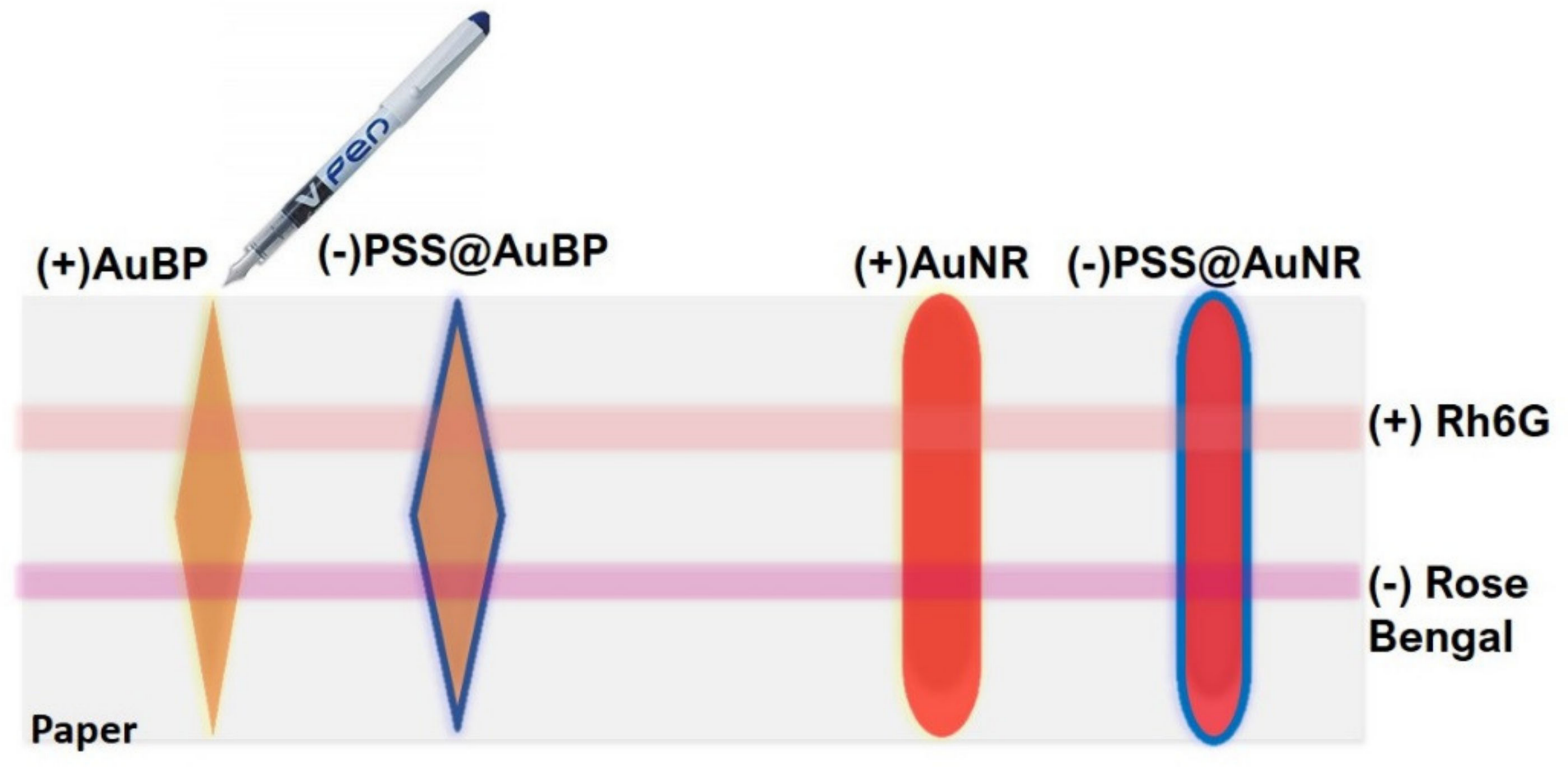
Illustration of the plasmonic calligraphy showing the design of different charged selective regions comprised of AuNRs and AuBPs (i.e. positively charged) and PSS-functionalized AuNRs and AuBPs (i.e. negatively charged) on the flexible paper-based substrate.

To evaluate the multiplex capabilities of our previously calligraphed plasmonic platform, one AuBPs and one AuNRs plasmonic line were coated with a layer of PSS (1 mg/mL) by using the same calligraphy approach, in particular, a ball point filled with the negatively-charged polyelectrolyte solution, thus obtaining different charge selective plasmonic regions consisting of two plasmonic lines of negatively-charged PSS-coated nanoparticles and two positively-charged CTAB-coated anisotropic plasmonic lines. After the PSS coating step, the plasmonic lines were thoroughly rinsed with ultrapure water to remove the unbound PSS. Afterwards, across the paper-based platform, we draw two isolated lines containing positively charged R6G and negatively charged RB analytes, perpendicularly to all four plasmonic lines, using the calligraphy pen filled with the fluorophore solution, as ink (see Scheme 1). 

### 2.5. Characterization Techniques

The UV-vis-NIR extinction spectra of the colloidal anisotropic nanoparticles were measured using a V-670 spectrophotometer (Jasco International, Tokyo, Japan,) with a 2 nm bandwidth and 1 nm spectral resolution. The size and morphology of the synthesized nanoparticles were determined using a TecnaiG2 F20 field emission transmission electron microscope (TEM, FEI Company, Hillsboro, OR, USA) operating at an accelerating voltage of 200 kV and equipped with a 4k CCD camera (Eagle, FEI Company, Hillsboro, OR, USA). The colloidal solutions were added dropwise onto a carbon film covered copper grid for TEM analyses. The surface charge of the nanoparticles was examined through zeta potential measurements using a Nano ZS90 Zetasizer analyser (Malvern Panalytical Ltd, Worcestershire, UK) equipped with a He–Ne laser (633 nm, 5 mW).

The optical properties of the as-calligraphed paper-based lines were collected using a portable USB 4000 optical UV-Vis spectrophotometer (Ocean Optics Inc., Largo, FL, USA), with a spectral resolution of 0.2 nm, coupled to an inverted optical microscope of an Axio Observer Z1 system (ZEISS, Jena, Germany) equipped with a halogen lamp (HAL 100) and a 10× ZEISS objective (NA = 0.45), through an optical fiber with a core diameter of 600 μm. The extinction spectra were recorded in absorption mode, using 5 accumulations and 50 ms integration time. The morphology and the distribution of the nanoparticles onto the cellulose fibers were investigated by scanning electron microscopy (SEM, FEI Company, Hillsboro, OR, USA) using a Quanta 3D FEG dual beam scanning electron microscope operating at an accelerating voltage of 30 kV. The plasmonic nanoplatforms were sputtered using a Q150R ES automatic sputter coater, in an argon atmosphere, with 5 nm gold layer for 10 min prior to the SEM investigation in order to inhibit charging, reduce thermal damage and improve the secondary electron signal required for the topographic examination in the SEM.

The SERS spectra of our plasmonic lines were recorded with a portable R3000CN spectrometer (Raman Systems, Singapore) equipped with a 785 nm diode laser, as an excitation source, coupled to a 100 μm optical fiber. The measurements were acquired at a laser power of 200 mW and a 10 s integration time.

Fluorescence measurements were recorded using a FP-6500 spectrofluorometer (Jasco International, Tokyo, Japan) with a 1 nm spectral resolution, equipped with a xenon lamp as excitation source, coupled to an epifluorescence accessory (EFA 383 module). Fluorescence spectra were collected in the wavelength range of 540–800 using a fixed excitation wavelength at 530 nm for R6G and 520 nm for RB.

## 3. Results

### 3.1. Optical/Morphological Characterization of the Colloidal and Calligraphed Anisotropic Nanoparticles

**Figure 1:**
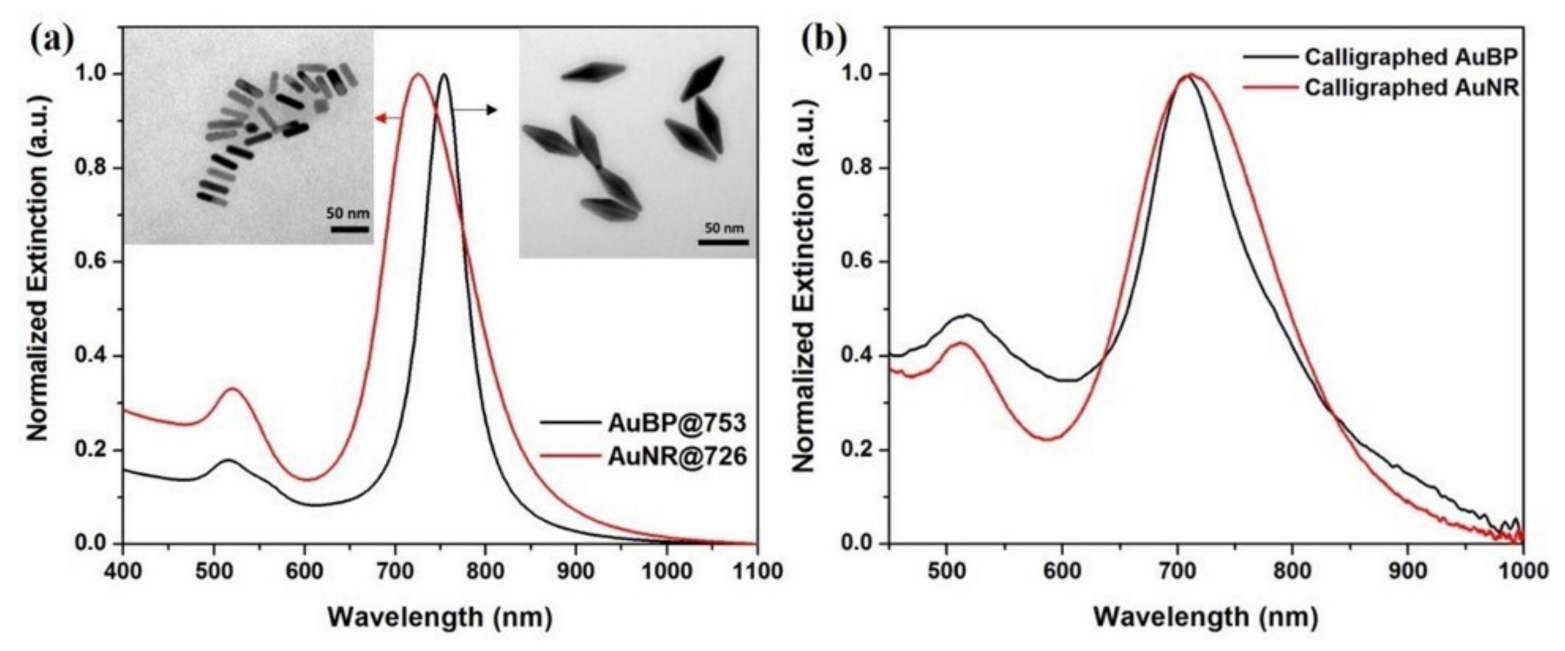
Normalized extinction spectra (**a**) of the colloidal AuBPs (black spectrum) and AuNRs (red spectrum) together with two representative TEM images and (**b**) collected directly from the calligraphed AuBPs (black spectrum) and AuNRs (red spectrum) lines onto the Whatman paper.

In view of the design an efficient plasmonic sensing array directly on a paper substrate comprising active lines with specific functionality, two different anisotropic plasmonic nanoparticles were selected and tested. Specifically, considering their strong electromagnetic field generated at their ends, also well-known as “intrinsic hot-spots” and the high tunability of the longitudinal LSPR (lLSPR), we, subsequently, synthesized colloidal AuBPs and AuNRs, as plasmonic inks, in order to present a similar plasmonic response after the immobilization step, aiming to evaluate and compare their efficiency. However, before their immobilization onto the cellulose fibres through the calligraphy-based procedure, the as-synthesized nanoparticles were first optically and morphologically characterized. Figure 1a presents the normalized extinction spectra of the colloidal AuNRs and AuBPs that will be later used as plasmonic inks to fabricate our plasmonic array on the paper substrate. Both UV-Vis-NIR spectra reveal the typical transversal LSPR (tLSPR) bands located at approximately 512 nm and the lLSPR bands with a maximum extinction located at 726 nm (red spectrum) and 753 nm (black spectrum), respectively, corresponding to AuNRs and AuBPs, respectively [30]. The successful formation of rod-shaped and diamond-like nanoparticles, with a good monodispersity in terms of shape and size, was proven by TEM imaging (insets Figure 1a). As a result, the dimensions of our nanostructures were evaluated by analyzing the corresponding TEM images using an image processing software (ImageJ), obtaining an average length x width of 38.5 nm × 12 nm for AuNRs and 68 nm × 23 nm for AuBPs. Zeta potential measurements were performed next to determine the surface charge of our CTAB-coated nanostructures, the obtained values are 39 ± 0.3 mV for AuNRs and 27 ± 0.2 mV for AuBPs (data not shown here). 

The as-synthesized positively-charged anisotropic NPs surface allows their easy integration into the 3D porous paper matrix formed by micro/nanoscale fibres interwoven together [31] via the electrostatic interaction between the hydroxyl groups of the cellulose fibres and the CTAB bilayer covering the NPs surface [32]. The extinction spectra collected from each traced plasmonic line onto the same Whatman paper prove that there are no spectral modifications, such as uncontrolled NPs’ aggregation or large lLSPR band broadening, except for the normal blue-shift due to the refractive index change from 1.333 (water) to 1 (air) [33], confirming—as such—the preserved optical properties of both AuNRs and AuBPs after the plasmonic calligraphy step (Figure 1b). Concretely, while the tLSPR bands maintains its position at around 512 nm, the lLSPR band undergoes a strong blue-shift of 15 nm for AuNRs (Figure 1b—red spectrum) and 46 nm for AuBPs (Figure 1b—black spectrum), respectively, both NPs exhibiting a longitudinal LSPR response at around 710 nm after immobilization. As such, AuBPs are more sensitive to the changes in the local dielectric properties of the microenvironment [34]. To note that the reproductivity of the as-calligraphed nanoparticles was also evaluated by collecting the plasmonic response in 3 different regions on the AuBPs line (Figure S1a) and on the AuNRs line (Figure S1b, while the stability of the calligraphed lines were investigated, finding that the designed nanoplatform is optically stable even after 5 months (Figure S2). 

The illustrative scanning electron microscopy (SEM) images prove the presence of the anisotropic NPs onto the paper fibres, seen as bright spots on the grey-colored paper matrix, with an individual distribution on the fibres without visible large-scale aggregation (Figure S3).

### 3.2. Bulk LSPR Sensing Performance of the Calligraphed Anisotropic Nanoparticles 

The sensing performance of the as-calligraphed plasmonic array onto the Whatman paper substrate was firstly evaluated by testing and comparing the bulk LSPR refractive index sensitivities (RIS; nm/RIU) and figures of merit (FOM) of the two types of the immobilized anisotropic nanoantennas. To do so, we prepared mixtures of water-glycerol with different values of the RI, spanning from n = 1.333 (water) to 1.473 (80% glycerol), which were then added dropwise onto the plasmonic lines, leading to a RI change of the surrounding environment of the nanoparticles. This change of the RI translates into a red-shift of the lLSPR band for both calligraphed AuNRs and AuBPs, which was monitored and plotted as a function of the RI value (Figure S4). Hence, judging from the experimentally obtained results, we conclude that the AuBPs line presents a better LSPR bulk sensing performance (i.e., FOM = 3.3; S = 262 nm/RIU and a coefficient of determination (R^2^) of 0.998) compared to the AuNRs plasmonic line ((i.e., FOM = 1.2; S = 137 nm/RIU and a coefficient of determination (R^2^) of 0.983) (details in Supplementary Materials). 

### 3.3. Calligraphed Plasmonic Array as Multiplex LSPR Charge-Selective Detection Scheme

**Figure 2:**
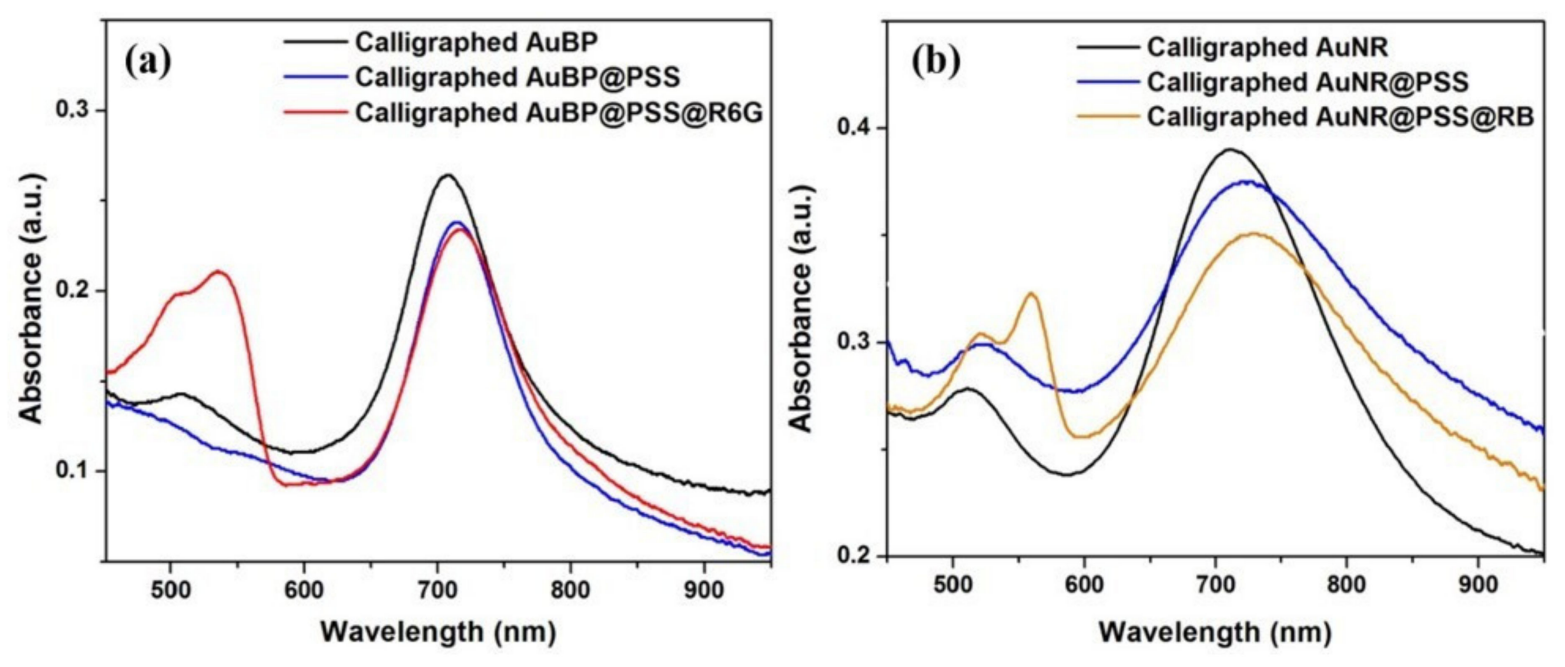
Extinction UV-Vis-NIR spectra of the as-calligraphed (**a**) AuBPs and (**b**) AuNRs lines (black spectra), functionalized with the negative PSS polyelectrolytes (blue spectra) and after the exposure to the cationic R6G (red spectrum) and anionic RB molecules (orange spectrum).

In order to design a well-isolated charge-selective plasmonic array with multiplex detection capabilities on the same paper support, we further employed the identical pen-on-paper approach to coat one AuBPs and AuNRs plasmonic line with a thin layer of negatively-charged PSS polyelectrolyte solution. After the controlled PSS deposition, the tLSPR and lLSPR bands of both anisotropic NPs types (Figure 2a,b—black spectra) undergo additional red-shifts, as a consequence of the increase of the refractive index of the environment surrounding the plasmonic NPs, thus demonstrating the successful adsorption of the PSS polyelectrolyte via electrostatic interaction (Figure 2a,b—blue spectra). Afterwards, onto the as-immobilized positively-CTAB charged plasmonic lines and as-engineered negatively-charged PSS plasmonic lines, we perpendicularly traced two isolated lines with fluorophores -used herein as target molecules—containing positively charged R6G and negatively charged RB analytes, respectively (previously described in Scheme 1). For exemplification, the successful selective functionalization step was, subsequently, validated by the direct electrostatic attachment of the cationic R6G on the negatively PSS coated AuBPs surface, inducing a bathochromic shift of the lLSPR of 7 nm, from 713 nm to 720 nm for the calligraphed AuBPs@PSS (Figure 2a—red spectrum), accompanied with the appearance of the electronic absorption band of the R6G molecules in the visible region. In fact, this lLSPR red-shift is attributed to the change of the surface dielectric constant, associated with the successful capturing of the R6G analyte to the calligraphed AuBPs@PSS line. A similar optical behavior was observed in the case of calligraphed PSS-functionalized AuNRs after the electrostatic interaction with the R6G molecules (Figure S5a—red spectrum). Contrarily, in the case of the negatively PSS-functionalized AuNRs line exposed to the anionic RB, although the characteristic electronic absorption band of the RB appears in the visible region of the electromagnetic spectrum, no LSPR red-shift was observed before (Figure 2b—blue spectrum) and after incubation (Figure 2b—orange spectrum), concluding that—in this case—the negatively-charged RB analyte is not captured by the negatively PSS-coated AuNRs. Anew, the negatively PSS-functionalized AuBPs line responds in the same manner, an electrostatic repulsion between these two negatively charged entities being revealed (Figure S5b).

### 3.4. Dual SERS-MEF “On/Off Switch” Sensing

**Figure 3:**
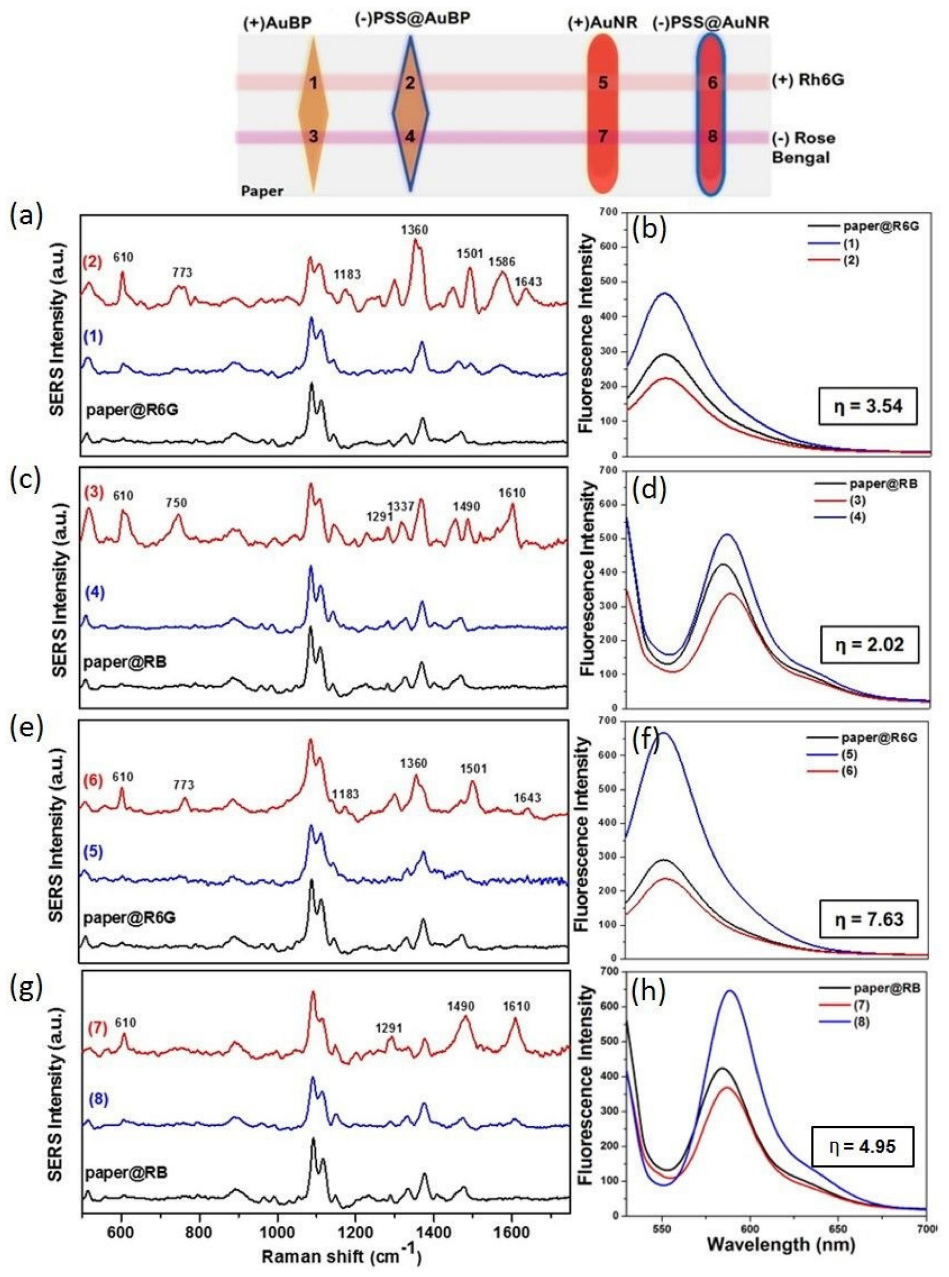
Validation of the “On/Off Switch" (nano)sensor illustrated on the top by complementary recording SERS detection (**a**,**c**,**e**,**g**-left panel) and MEF detection (**b**,**d**,**f**,**h**-right panel) spectra collected from the numbers (1–8) marked on the illustrative scheme compared to free calligraphed R6G (10^−4^ M) and RB (10^−4^ M) analyte onto the bare Whatman paper (black spectra) (i.e. near plasmonic line).

The plasmonic paper-based array, previously described as different charge-selective plasmonic regions engineered onto the same paper support, was next implemented as a dual optical “On/Off Switch” (nano)sensor for the complementary SERS/MEF detection of anionic and cationic molecules (herein, the RB and R6G target analytes). It is important to note, from the beginning, that the dual SERS/MEF spectra were collected from the same spots, which—for simplicity—were marked with numbers from 1 to 8 on the illustrative scheme represented below, and compared further to the free calligraphed R6G (10^−4^ M) and RB (10^−4^ M) analyte onto the bare Whatman paper (i.e. near plasmonic lines). The Raman and fluorescence spectra collected from all charge-selective calligraphed plasmonic lines exposed to cationic R6G and anionic RB analytes are presented in Figure 3, revealing—as a first observation—that different active domains were effective at detecting different charged molecules.

As a result, the negative PSS-coated AuBPs line (Figure 3a, red spectrum) and negative PSS-coated AuNRs line (Figure 3e—red spectrum) were able to electrostatically capture and detect the cationic R6G molecules demonstrated by the assignment of their characteristic vibrational Raman bands corresponding to located at 610 cm^−1^ (C–C–C ring in-plane vibration), 773 cm^−1^ (C–H out-of-plane bending), 1183 cm^−1^ (C–H and N–H bending of xanthene ring), 1360/1507/1648 cm^−1^ (aromatic C–C stretching) [35,36], and summarized also in Table 1.

Just for exemplification, the reproducibility of the recorded SERS signal of the cationic R6G molecules electrostatically captured by the negative PSS-coated AuNRs line was tested and presented in Figure S6.

As reference, the corresponding Raman spectrum was collected on the calligraphed free R6G molecules of same concentration (10^−4^ M) onto the bare Whatman paper (near the plasmonic line) (Figure 3a,e—black spectra), while the Raman spectra of the bare Whatman paper itself, calligraphed with AuBPs, AuNPs or calligraphed with a higher concentration of R6G is reported in the Supplementary Information (Figure S7—blue spectrum). The selective SERS detection of the cationic target molecule by the negatively PSS-functionalized AuBPs or AuNRs, by the excitation of the longitudinal LSPR band using a 785 nm laser line, clearly proves a strong electrostatic interaction between R6G and PSS, concluding that the analytes are placed in close contact with the metal, where the electromagnetic enhancement is still operational, promoting hence SERS. Considering that R6G presents the absorption band in the visible range, it should be mentioned, herein, that no resonant Raman effects are supposed to contribute to the overall SERS enhancement for the employed 785 nm excitation laser. In parallel, the fluorescence emission of R6G molecules collected exactly in the same spot as the SERS spectra (Figure 3b,f—red spectra) is quenched compared to the emission of R6G on bare paper, as control (Figure 3b,f—black spectra), due to the non-radiative losses in the metal, concluding that this selective electrostatic interaction is beneficial for SERS, but unfavourable for MEF sensing. 

Similar results were obtained in the case of the anionic RB capture by the positively charged CTAB layer that covers both AuBPs and AuNRs calligraphed on paper. Figure 3c,g—red spectra prove that the anionic molecules were selectively detected by both positively charged CTAB-coated nanostructures, SERS spectrum possessing the characteristic bands at 610 cm^−1^ (C–C–C ring in-plane vibration), 750 cm^−1^ (C–Cl stretching), 1337 cm^−1^ (C–C stretching in ring), 1490 cm^−1^ (C=C asymmetric stretching in ring), 1547 cm^−1^ (C–C stretching in ring), 1612 cm^−1^ (C=C symmetric stretching in ring) [16,37] as predicted in Table 1. As reference, the corresponding Raman spectra were collected on the calligraphed free RB molecules (10^−2^ M concentration) onto the bare Whatman paper (Figure S7—brown spectrum).

As in the previous case of the cationic R6G bound to the PSS-coated nanoparticles, herein, the interaction between the anionic RB and positively charged plasmonic lines is evaluated—in parallel—by fluorescence sensing, observing the same behavior when anionic molecules are electrostatically grafted on the plasmonic surface, namely, the quenched emission of our RB molecules (Figure 3d,h—red spectra) compared to the reference spectra (i.e. RB on bare paper) (Figure 3d,h—black spectra).

On the other hand, when the target analytes have the same charge as the plasmonic lines (i.e., RB on PSS-coated nanoparticles and R6G on CTAB-coated nanoparticles), the test domains exhibited very weak or no Raman fingerprints for R6G or RB (Figure 3—blue spectra). The absence of the vibrational bands in both cases is caused by the electrostatic repulsion between the cationic/anionic molecules and the surface chemistry of NPs or PSS-coated plasmonic nanoparticles. Remarkably, since there is no direct interaction between the fluorophores and nanoparticles, proven by LSPR and Raman results, the anionic RB and cationic R6G molecules are not positioned on the surface of the nanoparticles, but rather in their close vicinity, which translates to an enhanced emission for both R6G and RB relative to their emission on bare filter paper (Figure 3—right panel, blue spectra), by exciting both the tLSPR of the NPs and electronic absorption band of the fluorophores. To conclude, in this case, the electrostatic repulsion keeps the molecules at the optimal distance from the Au surface to prevent fluorescence quenching and promote, consequently MEF, but far enough to inhibit the SERS performance of the charge-selective plasmonic lines, proving, as such, the feasibility of our nanoplatform to operate as a complementary “ON/OFF Switch” nanosensor for specific anionic/cationic detection. We also point out that multiple Raman and fluorescence spectra were measured on other substrates as well, fabricated using the same preparation protocol of charged isolated plasmonic lines, obtaining the same results, which ensures the high reproducibility of our nanoplatform.

Furthermore, as a general conclusion regarding the SERS detection, a better performance of the calligraphed AuBPs line as SERS sensing (nano)antennas compared to calligraphed AuNRs line was noticed for both cationic R6G and anionic RB molecules. Specifically, by performing a systematic comparison of the SERS detection capability of these two types of elongated nanoparticles having a similar plasmonic response at around 710 nm, we observed that the AuBPs line is able to generate a much stronger SERS signal than the AuNR line (Figure 3). This experimentally obtained result is not surprising considering that AuBPs present a higher intrinsic field enhancement generated at their sharp tips compared with their nanorod counterparts [38].

Second, regarding the MEF detection, we conclude that when the target analytes are electrostatically repulsed by the NP’s surface, the SERS detection was compromised, but the distance from the metal is still operational to generate MEF by exciting both the transversal LSPR of the NPs and electronic absorption bands of the dyes. At this point, we should point out that the *apparent* fluorescence enhancement measured from a spot in the presence of nanoparticles integrates both non-altered fluorescence collected from molecules located outside of any interaction with nanoparticles (“distant molecules”) and enhanced fluorescence collected from molecules located in the high field generated by nanoparticle excitation (“close molecules”) which in fact promote MEF. Alternatively, the apparent decrease of fluorescence when measured from a spot which exhibit high SERS signal is consequent with non-altered fluorescence collected from all distant molecules, as the molecules in contact with metal exhibit quenched emission and, in fact, promote SERS. To evaluate the true amplification factor (*ƞ*), a correction to the recorded data has been applied (see details in Supplementary Materials). More importantly, a different MEF detection performance between these two types of elongated NPs was also observed, the AuNRs proving a superior MEF efficiency by recording a maximum of 7.63-fold enhancement for R6G and a 4.96-fold enhancement for RB, values also mentioned in Figure 3—right panel for each specific case. The highest recorded MEF factors for AuNRs are due to better overlapping of the tLSPR with the absorption band of target dyes. 

In conclusion, by employing the pen-on-paper approach we demonstrate the facile fabrication of the charge-selective active array domains and—consequently—control the SERS and MEF detection of differently charged target analytes of interest.

## 4. Conclusions

To summarize, in the current paper, a novel dual optical "ON/OFF Switch nanosensor was designed by taking advantage of the plasmonic calligraphy approach—as a simple, yet powerful tool—to miniaturize highly efficient plasmonic lines and obtain, consequently, a multiplexed charge selective complementary dual SERS/MEF array for the detection of different anionic or cationic target analytes. Concretely, two different elongated plasmonic transducers, namely AuBPs and AuNRs, were used as inks in a common ball-point pen to create well-isolated plasmonic lines. We have demonstrated the feasibility of the approach for multiplexed sensing using two target analytes, the anionic RB and cationic R6G molecules. By monitoring the lLSPR red-shift upon exposure to differently charged molecules, we were able to confirm the presence or absence of each analyte on the specific charged test domain. The electrostatic attachment of R6G and RB to the AuBPs or AuNRs allows the identification their molecular Raman fingerprint via SERS, while consequently being responsible for the quenching of the fluorescence emission. Moreover, due to electrostatic repulsion, the fluorophores remain in the close vicinity of the plasmonic nanostructures, ensuring thus the optimal distance for MEF and, implicitly, no SERS detection, proving hence the feasibility of our design nanoplatform to operate as a complementary “ON/OFF Switch” nanosensor for specific anionic/cationic detection. Plasmonic calligraphy serves as a powerful tool to fabricate miniaturized plasmonic-substrates with specific functionalities, great stability and high reproducibility, thus proving themselves as promising biosensing platforms for the implementation in more complex applications/ in real-world samples.

## Figures and Tables

**Table 1:** Vibrational mode assessments for SERS bands recorded in Figure 3.

Analyte	SERS Signal (cm^−1^)	Band Assignment
**R6G**	610 773 1183 1360 1501 1586 1643	C–C–C ring in-plane vibration C–H out-of-plane bending C–H and N–H bending of xanthenes ring aromatic C–C stretching aromatic C–C stretching aromatic C–C stretching aromatic C–C stretching
**RB**	610 750 1291 1337 1490 1547 1612	C–C–C ring in-plane vibration C-Cl stretching CCC deformation in ring and C–H deformation C–C stretching in ring C=C asymmetric stretching in ring C–C stretching in ring C=C symmetric stretching in ring

## Supplementary Materials

The following are available online at https://www.mdpi.com/2079-4991/10/6/1025/s1, Figure S1: The UV-Vis-NIR extinction spectra collected in 3 different regions on the calligraphed AuBPs line (a) and the calligraphed AuNRs line (b) onto the Whatman paper, Figure S2: The UV-Vis-NIR extinction spectra of the calligraphed AuBPs (a) and AuNRs (b), respectively, recorded immediately after the nanoparticle’s immobilization onto the Whatman paper (black spectra) and after 5 months (red spectra), Figure S3: Illustrative SEM images of the bare Whatman filter paper before (as control) and after the calligraphy step using colloidal AuBPs and AuNRs inks, Figure S4: Dependence of the longitudinal LSPR position as a function of the bulk refractive index for both calligraphed AuBPs (black line) and AuNRs (red line), Figure S5: Extinction UV-Vis-NIR spectra of the as-calligraphed (a) AuNRs and (b) AuBPs lines (black spectra), functionalized with the negative PSS polyelectrolytes (blue spectra) and after the exposure to the cationic R6G and anionic RB molecules (red spectra), Figure S6: SERS spectra of cationic R6G molecules electrostatically captured by the negative PSS-coated AuNRs line platform collected from different sites on the spot marked with number 3 on the illustrative scheme represented in Figure 3, Figure S7: The Raman spectra of the bare Whatman paper itself (black spectrum), calligraphed with AuBPs (red spectrum), AuNPs (orange spectrum) or calligraphed with a higher concentration of free R6G (blue spectrum) and free RB (brown spectrum). Excitation: portable 785 nm laser line.
