# Supplementary material for: Calligraphed Selective Plasmonic Arrays on Paper Platforms for Complementary Dual Optical “ON/OFF Switch” Sensing
Source: Nanomaterials (Basel). 2020 May 27;10(6):1025. doi: 10.3390/nano10061025 (PMC7352805; doi:10.3390/nano10061025)
Supplement: Supplementary file 1 [file nanomaterials-10-01025-s001.pdf]

Supplementary Information

Calligraphed Selective Plasmonic Arrays on Paper Platforms for Complementary Dual Optical "ON/OFF Switch" Sensing

Laurentiu Susu [1,2], Andreea Campu [1,2], Simion Astilean [1,2] and Monica Focsan [1,*]

[1] Nanobiophotonics and Laser Microspectroscopy Center, Interdisciplinary Research Institute on Bio-Nano-Sciences, Babes-Bolyai University, Treboniu Laurean No. 42, Cluj-Napoca 400271, Romania; susulaurentiu@yahoo.com (L.S.); andreea.campu@gmail.com (A.C.); simion.astilean@phys.ubbcluj.ro (S.A.)

[2] Biomolecular Physics Department, Faculty of Physics, Babes-Bolyai University, M Kogalniceanu No. 1, Cluj-Napoca 400084, Romania

* Correspondence: monica.iosin@phys.ubbcluj.ro; Tel.: +40-264-454554 (ext 116)

Received: 24 April 2020; Accepted: 25 May 2020; Published: date

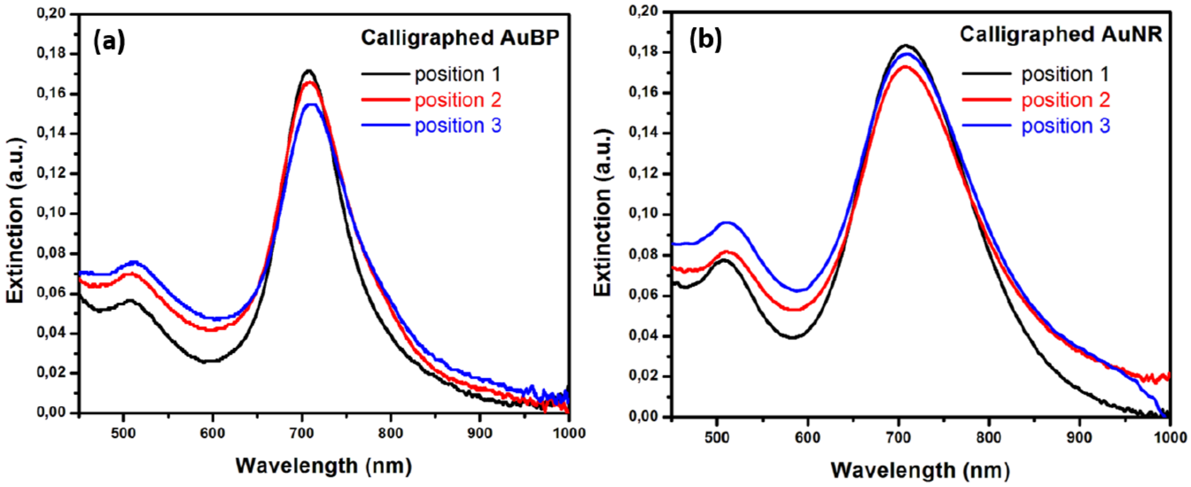

Figure S1. The UV-Vis-NIR extinction spectra collected in 3 different regions on the calligraphed AuBPs line (**a**) and the calligraphed AuNRs line (**b**) onto the Whatman paper.

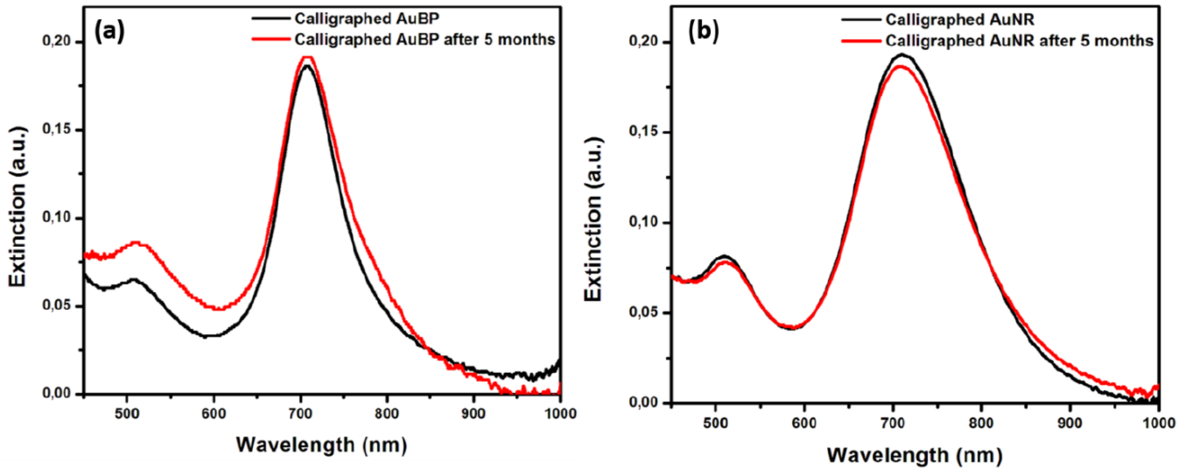

Figure S2. The UV-Vis-NIR extinction spectra of the calligraphed AuBPs (**a**) and AuNRs (**b**), respectively, recorded immediately after the nanoparticle's immobilization onto the Whatman paper (black spectra) and after 5 months (red spectra).

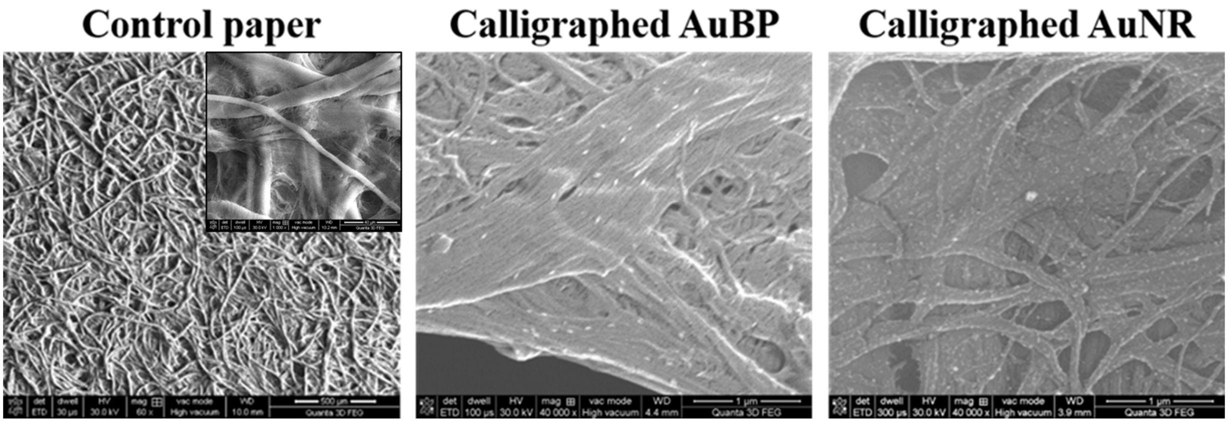

Figure S3. Illustrative SEM images of the bare Whatman filter paper before (as control) and after the calligraphy step using colloidal AuBPs and AuNRs inks.

Bulk LSPR sensing performance of the calligraphed anisotropic nanoparticles

The bulk refractive index sensitivities (RISs) of the calligraphed NPs were determined from the slopes of the linear regressions presented in Figure S2, obtaining values of 264 nm/RIU for AuBPs and 150 nm/RIU for AuNRs. As a consequence, the calligraphed AuNRs results in a lower figure of merit (FOM) than AuBPs, this parameter being defined as:

$$FOM = \frac{bulk\ RIS}{FWHM}$$

where FWHM represents the fullwidth half-maximum of the l LSPR band [1,2]. In fact, it is clear from the above equation that the FOM values decrease for the calligraphed AuNRs due to the diminish of the bulk RIS combined with a small increase in the FWHM. So, judging from the experimentally obtained results, we can conclude that the AuBPs line presents a better LSPR sensor' performance compared to the AuNRs plasmonic line

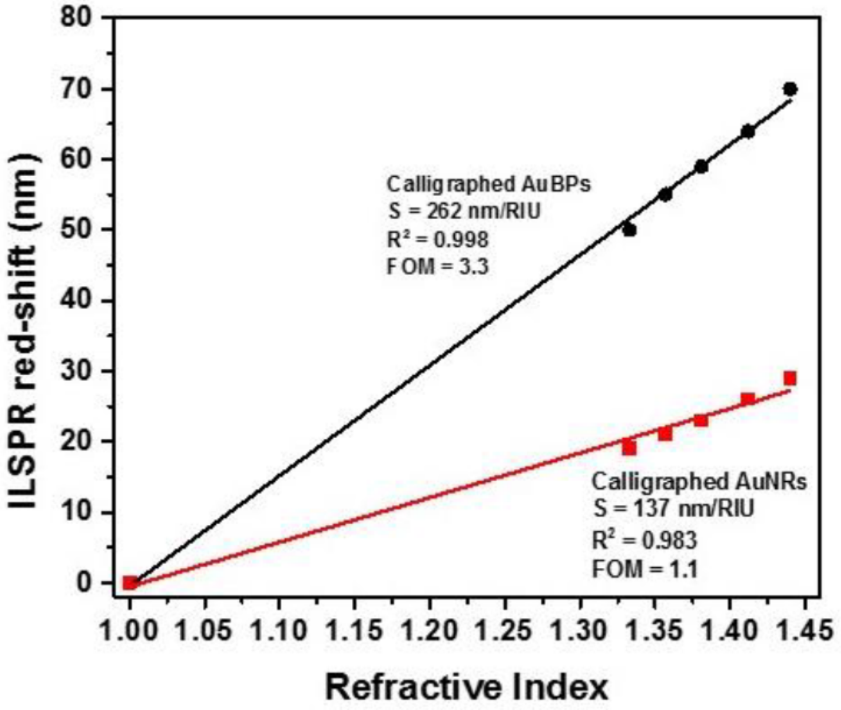

Figure S4. Dependence of the longitudinal LSPR position as a function of the bulk refractive index for both calligraphed AuBPs (black line) and AuNRs (red line).

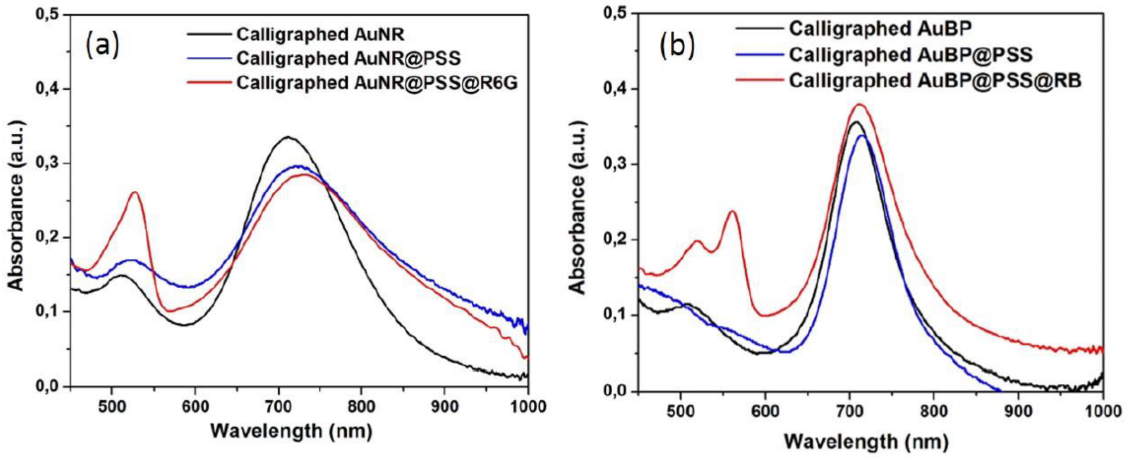

Figure S5. Extinction UV-Vis-NIR spectra of the as-calligraphed (**a**) AuNRs and (**b**) AuBPs lines (black spectra), functionalized with the negative PSS polyelectrolytes (blue spectra) and after the exposure to the cationic R6G and anionic RB molecules (red spectra).

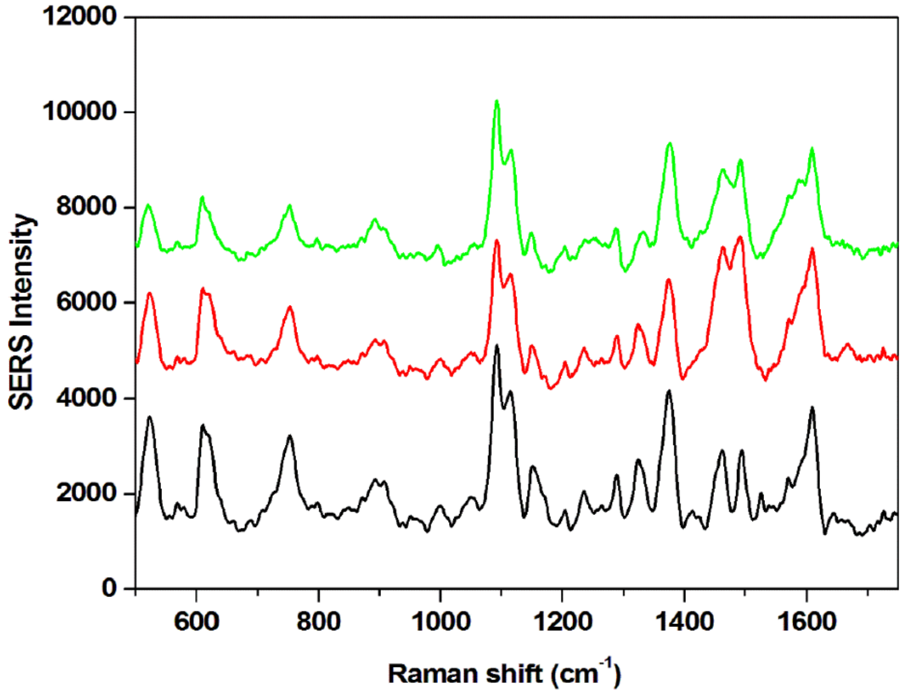

Figure S6. SERS spectra of cationic R6G molecules electrostatically captured by the negative PSS-coated AuNRs line platform collected from different sites on the spot marked with number 3 on the illustrative scheme represented in Figure 3.

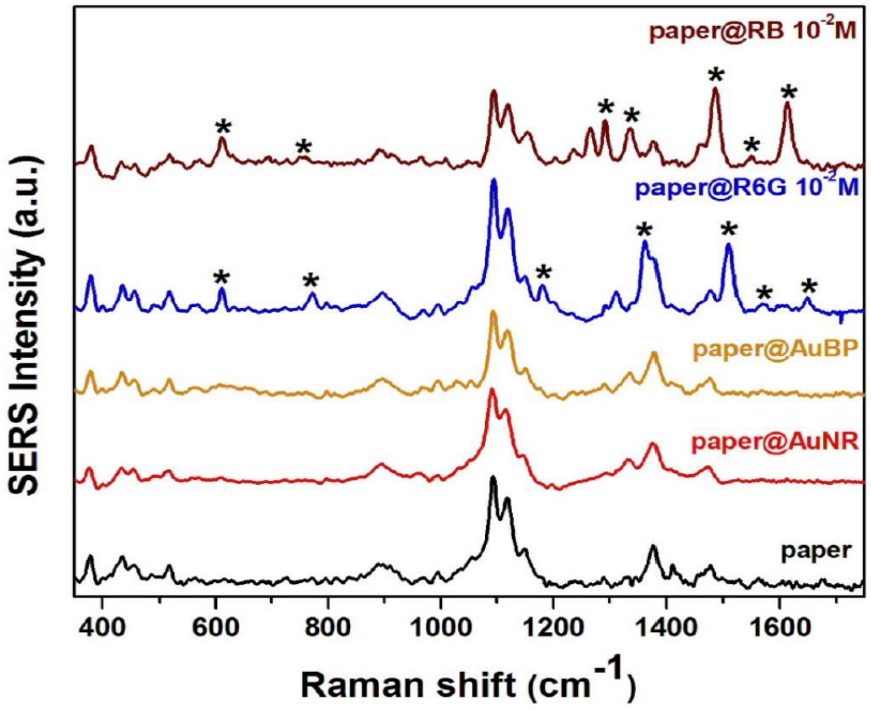

Figure S7. The Raman spectra of the bare Whatman paper itself (black spectrum), calligraphed with AuBPs (red spectrum), AuNPs (orange spectrum) or calligraphed with a higher concentration of free R6G (blue spectrum) and free RB (brown spectrum). Excitation: portable 785 nm laser line.

Evaluation of the fluorescence amplification factor

The fluorescence intensity, I_F is proportional to the amount of light absorbed and the fluorescence quantum yield, Φ:

$$I_{\mathrm{F}} = \alpha \times I_{\mathrm{i}} \times \Phi \times (\varepsilon \times b \times c)$$

where, α is a proportionality constant attributed to the instrument, I_i is the incident light intensity, ε is the molar absorptivity, b is the optical path length and c is the concentration. For simplicity, we define k as the following product:

$$k = \alpha \times I_{\mathrm{i}} \times \Phi \times (\varepsilon \times b \times c)$$

since the fluorescence measurements were conducted in the same experimental conditions.

In our case, the fluorescence intensity of the two fluorophores (i.e. R6G and RB) can be defined using the following equation:

$$I_0 = k \times N_0 \quad (1)$$

where I_0 is the fluorescence intensity measured in normal fluorescence spectra of the fluorophore (observed in the absence of the quencher) and N_0 are the total number of free fluorophore molecules.

In the presence of quencher I_q, the fluorescence intensity equation is written as:

$$I_{\mathrm{q}} = k \times (N_0 - N_{\mathrm{q}}) \quad (2)$$

where the total number of molecules, which are electrostatically attached to the surface of the nanoparticles, N_q, are taken into account. Thus, the amplified emission intensity I_A can be expressed as:

$$I_{\mathrm{A}} = k \times (N_0 - N_{\mathrm{A}}) + \eta \times k \times N_{\mathrm{A}} \quad (3)$$

where N_A are the total number of molecules which are in the close vicinity of the nanoparticles and η represents the fluorescent enhancement factor. We admit that $N_q = N_{A,}$ meaning that the number of the nanoparticle-attached molecules is equal to the number of molecules having their emission amplified, this condition leads to the following form of Equation (3):

$$I_A = k \times (N_0 - N_{\mathrm{q}}) + \eta \times k \times N_{\mathrm{q}} \quad (4)$$

Taking into consideration, that the first product corresponds to the fluorescence intensity in the presence of the quencher, equation (4) becomes:

$$I_{\mathrm{A}} = I_{\mathrm{q}} + \eta \times k \times N_{\mathrm{q}} \quad (5)$$

Finally, by introducing the florescence intensity of the fluorophores, we obtain the expression of the amplified emission intensity as:

$$I_{\mathrm{A}} = I_{\mathrm{q}} + \eta \times (I_0 - I_{\mathrm{q}}) \quad (6)$$

Thus, the enhancement factor η can be extracted as follows:

$$\eta = \frac{I_{\mathrm{A}} - I_{\mathrm{q}}}{I_0 - I_{\mathrm{q}}}$$

The calculated η factors for the fluorescence enhancement of R6G in the presence of CTAB-coated nanoparticles and RB in the presence of PSS-coated lines are presented in the emission spectra in Figure 2-right panel in the manuscript.

References:

1. Otte, M.A.; Sepulveda, B. Figures of Merit for Refractometric LSPR Biosensing. *In Nanoplasmonic Sensors*, Springer: New York, NY, USA, 2012, pp. 317–331.
2. Doiron, B.; Mota, M.; Wells, M.P.; Bower, R.; Mihai, A.; Li, Y.; Cohen, L.F.; Alford, N.M.; Petrov, P.K.; Oulton, R.F.; et al. Quantifying Figures of Merit for Localized Surface Plasmon Resonance Applications: A Materials Survey. *ACS Photonics* **2019**, *6*, 240–259.
